# Determinants of Junk Food Consumption Among Adolescents in Pokhara Valley, Nepal

**DOI:** 10.3389/fnut.2021.644650

**Published:** 2021-04-08

**Authors:** Suraj Sujan Bohara, Kanchan Thapa, Laxman Datt Bhatt, Shankar Singh Dhami, Shreejana Wagle

**Affiliations:** ^1^Faculty of Health Science, School of Health and Allied Sciences, Pokhara University, Kaski, Nepal; ^2^Independent Public Health Researcher, Kathmandu, Nepal; ^3^Department of Healthcare Management, National Open College, Pokhara University, Kaski, Nepal

**Keywords:** adolescents, food preferences, junk food consumption, nutrient, non-communicable disease

## Abstract

**Background:** Junk food consumption and its consequences has become a major public health concern globally because of its deteriorating health consequences and surging prevalence. Though its adverse health consequences are widely prevalent in all age groups, children and adolescents are more at risk. It may lead to obesity and act as a risk factor for different non-communicable diseases (NCD's) like heart diseases, cardiovascular disease, cancer, hypertension, diabetes, etc. This study was carried out to explore the junk food consumption and its associated factors among adolescent students.

**Methods:** A cross-sectional study was conducted among 538 adolescent students of Kaski district, Nepal. We used a stratified proportionate sampling technique to recruit the participants. A self-administered questionnaire was used for data collection. Descriptive and bivariate statistical analysis was performed. The odds ratio was computed to test the association.

**Results:** The study found that more than half of the participants (60.30%) consumed junk foods over the last 30 days, more prevalent among public school participants (65.1%) followed by participants of private school (56.3%). More than half of the participants consumed salty snacks (58.7%) followed by sweets (57.5%). The time of consumption was found to be higher together with friends (83.9%). Similarly, it was consumed more while the participants were on a trip (70.1%). Consumption of junk foods was significantly associated with public school (OR: 1.44, CI = 1.01–2.06), single family (OR: 1.46, CI = 1.01–2.10), living with parents (OR: 1.64, CI = 1.03–2.63), while on travel (OR: 1.99, CI = 1.33–2.98), while reading (OR: 2.01, CI = 1.16–3.47), at home (OR: 2.20, CI = 1.53–3.16), at school (OR: 2.86, CI = 1.98–4.12), friends' influence (OR: 2.01, CI = 1.37–2.94), and junk food availability at home (OR: 1.92, CI = 1.33–2.76).

**Conclusion:** Consumption of junk foods among adolescent students was remarkably high in both public school and private school adolescents. Regardless of adequate knowledge on harmful consequences of junk foods, school-going adolescents are consuming junk foods due to its easy availability and ready-to-use packaging. The government of Nepal should strictly standardize and regulate advertising policies and extravagant health claims advertised by junk food manufacturers. An appropriate intervention targeted to adolescents to improve food behaviors is recommended.

## Introduction

Junk foods are defined as foods that are readily available, usually inexpensive, and having less nutrient value. These foods contain more calories, more salt, have a higher content of saturated fat, and contain less iron, calcium, and dietary fiber. Common junk foods include fast food, carbonated drinks, chips, desserts, chocolates, etc. (1).

Globally, junk foods are popular stuff, and consumption is increasing constantly. Traditional foods have been nearly replaced by food items that can be found in a state of ready to eat, in canned form, and preserved for a longtime (2). The consumption of such foods has peaked in developed countries; however, there is an increasing trend in the developing countries of the world (3). In South Asian countries, there is a clear rising trend of such junk food consumption (4, 5). Despite established evidence of the negative impacts of junk foods on the human body, the consumption of junk foods is popular among youngsters. Such consumption may lead to a high prevalence of obesity, diabetes mellitus, hypertension, and coronary heart disease (6).

It is estimated that 16 million (1.0%) disability-adjusted life years (DALYs) lost and 1.7 million (2.8%) of worldwide mortality have been attributed to inadequate consumption of vegetables and fruits (7). Despite the socioeconomic condition of the family, junk food consumption has been emerging worldwide due to quick consumption, ready to eat, inexpensive, and of good taste. Such foods have been found prepared using low-quality ingredients such as refined grains, added sugar, and fats, despite nutritious ingredients (8). Fast foods have high sodium salt, which is often used as a preservative to make the foods more flavorful and satisfying. Such foods attract more people especially children and adolescents (9).

Increased junk food consumption among all age groups and more common to young adults is an emerging public health challenge with global prevalence of around 70%. Rapidly changing dietary practices and an increasing sedentary lifestyle predispose to obesity-related non-communicable diseases, including insulin resistance diabetes, neurodegeneration, and psychological changes, stroke, headache/precipitation of migraine, the metabolic syndrome, adult-onset diabetes, non-insulin-dependent diabetes, coronary artery diseases, polycystic ovarian syndrome, non-alcoholic fatty liver disease, cancers, and autoimmune disorders and site-specific neoplasms, both in children and in adults. Recent data show that obesity-related non-communicable diseases are increasing in many developing countries with cross-sectional and secular trends of childhood obesity globally and more prevalent to developing countries (10, 11).

Obesity and overweight has increased many fold in Asia, and it is becoming more alarming in recent years. Countries of the World Health Organization (WHO) South East Asia Region are facing an epidemic of diseases associated with obesity such as diabetes and cardiovascular disease (CVD). Various studies had shown a rising prevalence of obesity among children due to their risky behaviors and dietary patterns (12).

Despite facts known among adolescents in Nepal, there is a gap to explore food consumption patterns and association with obesity. Since adolescents account for a quarter of the country's population, there should be special strategies to think about their current nutritional status (13). A recent study from Kaski district depicts 8.1% prevalence of overweight and obesity among adolescents (14). Another study conducted in the Kaski district of Nepal shows that the obesity prevalence among adolescents is 3.3% (15). Risky behaviors such as unusual time of sleeping, tobacco and substance abuse, watching television for a longer time, consuming low dietary foods and fruits, along with insufficient physical activities are found to be more prevalent in the Kaski district, which are leading to more risk of deviating health condition of adolescents (16–18). About six among 10 deaths are found to be caused by NCD in Nepal; among them, nearly a quarter of these have been caused by cardiovascular diseases (19). So, we are in a better position to think about food habits among adolescents to prevent further complications.

There is limited evidence to identify the magnitude of the junk food prevalence and factors promoting its consumption. We explored the status of junk food consumption and its associated factor among the adolescents in the Kaski district of Nepal. Findings of this study are expected to be a primary step toward planning multipronged strategies to address the growing health hazard and protecting children and adolescents from the long-term ill health effects of junk foods. The study results will have policy implications for adolescents to plan, prevent, and control junk foods, obesity, and other health complications.

## Methods

### Study Design

An analytical cross-sectional study was conducted among selected school-aged adolescents in the Kaski district of Nepal from July 2017 to December 2017.

### Study Areas

The study was conducted in 54 private and 47 public schools of Pokhara metropolitan (formerly Lekhnath municipality), Kaski district, Nepal. Kaski district is one of the largest cities of Gandaki Province, which comprises a total of 492,098 population, which is 1.86% of the national estimated population. The district has 46.3% adolescents, and it ranks third on literacy rate (82.38) and Human Development Index (0.576) with a poverty gap of 0.79 in the district (20).

### Study Population

This study recruited school-going adolescents studying in grades 11 and 12 of selected schools in Pokhara metropolitan. The students of school having only girl's cohort or boy's cohort, physically challenged, and visually impaired students were excluded from this study.

### Sample Size Estimation

Sample size was calculated by using the following formula: sample size (*n*) = Z^2^pq/d^2^ [Z = 1.96 for 95% confidence interval, P = proportion of population with certain characteristic, q = proportion of population without certain characteristic, and d = allowable error (0.05 for 5%)].

Thus, sample size was computed to be 274 and after adjusting the non-response rate to 5%, the sample size was 290. Since the study included participants of both public and private schools at a ratio of 5:6, therefore, by adjusting the proportion ratio of 5:6, the sample size for public schools was 245 and private school was 290. Therefore, the final sample size of this study was 245 + 290 = 535 (Sample size calculation described in the Table 1).

**Table 1:** Sample size calculation table.

**Variables**	**Proportion or mean**	**Sample size**	**Reference country**
Junk food consumption	[23.26%]	274	Brazil (21)
Junk food available at home	[22.1%]	265	US (22)
Average knowledge of the harmful effect of junk	[18.33%]	230	India (23)
Junk food advertisement	[22.2%]	265	Nepal (24)

### Sampling Technique

**Figure 1:**
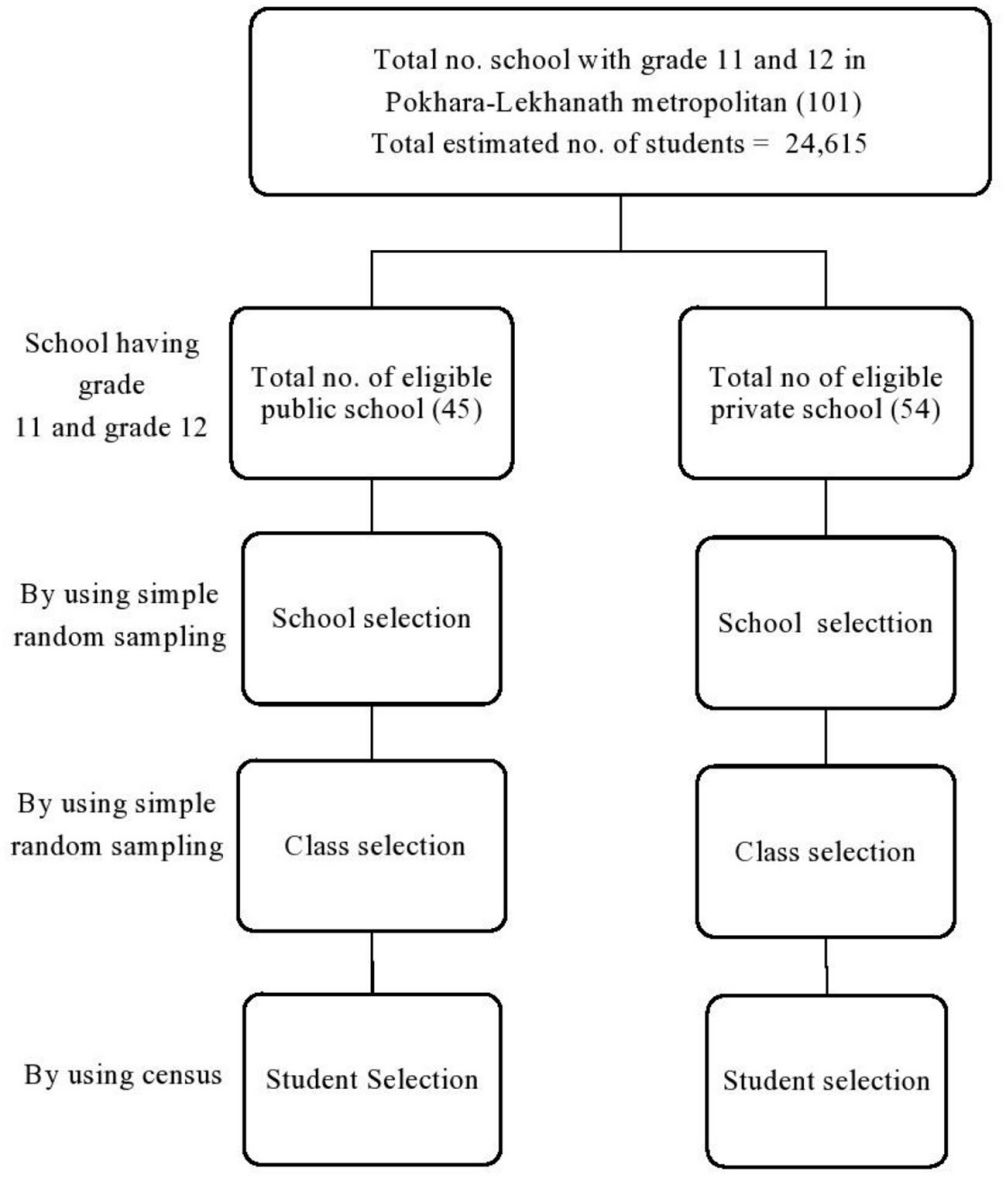
Flow diagram of sampling technique.

A stratified proportionate sampling method was used to select participants. We designed a disaggregated sampling frame for public and private secondary schools of Kaski district, and the required proportion (5:6) was taken from the type of schools and the required participants from them Sampling technique is described in the diagram (Figure 1).

### Data Collection Method

The data were collected using the standard self-administered questionnaire among the adolescent students during their school day, and a 30-min time was allocated to complete the questionnaire. Written informed consent was taken from the teacher on behalf of the participants, and the teacher was informed about the student's volunteer participation before the consent grant on behalf of the student. The purpose of the study, confidentiality of their answer, and there is nothing like right or wrong were briefed before data collection. Seven-day dietary recall methods were used to report their recent week dietary pattern. The data were collected from October 29 to November 8, 2017 in Pokhara metropolitan of Kaski district.

### Data Collection Tool

A self-administered questionnaire was used for data collection. The questionnaire was pretested and validated before the final data collection. Extensive literature review was done, and a tool was developed reviewing similar literature, which reports the factors that accelerate junk food consumption. The tool was initially developed in English version and translated into Nepali. The linguistic validation and context validation was checked and recommended by nutrition experts and academic professors prior to pretesting.

### Variables

#### Independent Variables

The independent variables are age, sex, parents' education, living status, family type, parents' occupation, advertisement, convenience, peer influence, availability of junk food, and knowledge about the health effect of junk food consumption.

#### Dependent Variable

The dependent variable is junk food consumption.

### Operational Definitions

#### Adolescent Student

Students of grade 11 and grade 12 who are not older than 19 years of age.

#### Junk Food

Junk food includes instant noodles, biscuits, cookies, chip lays, chocolates, cake, ice cream, chow mien, Mo: Mo, samosa, soft drinks, Coke, Pepsi, Fanta, burgers, pizza, canned foods, fried potatoes, meat products, etc.

#### Junk Food Consumption

A student consumes at least one item of junk food for 3 days or more within the last 7 days.

### Frequency of Consumption

**Mostly**

It is the time that is more than 15 days in a month and more than 4 days in a week.

**Often**

It is the time that is 5–15 days in a month and 2–4 days in a week.

**Sometimes**

It is the time that is <5 days in a month and <2 days in a week.

#### Place of Consumption

It is the place such as home, school, friend's home, etc., where the individual adolescent student consumes any type of junk food.

#### Living Situation

An adolescent's current living status with parents, relatives, friends, in a hostel, alone, or in any other condition.

#### Peer Influence

Peer influence is the perceived impact of peer groups, their encouragement, involvement, facilitation, and role modeling for other friends.

#### Knowledge

Participants who answered the right option among the given three questions in the questionnaire that were classified as adequate knowledge regarding the harmful effects of junk foods and others who did not answer right among the three were classified as inadequate knowledge.

### Validity and Reliability

The study tool was reviewed by the researcher and nutrition expert to maintain the validity and reliability of the data collection. Pretesting was done among the 50 students of Pokhara valley, and all the required revision based on pretesting was done on the data collection tool. Pretesting samples were excluded in the final enumeration. Based on the findings of pre-testing, necessary modification on the tool was done prior to data collection. The test of normality was done for those nonparametric distributions, and the median values were computed and presented in the *Results* section.

### Data Management and Analysis

Data were entered in Epi-data and imported into SPSS version 22. A descriptive and interferential analysis of data was performed. Odds ratio (OR) with a value greater than 1.00 is considered as significant. Similarly, chi-square testing was done. Those variables having a p-value less than 0.05 mentioned for risk ratio (RR). Thus, the computed RR yield odds ratio (OR) at 95% confidence interval and 5% level of significance.

## Results

Information about the demographic characteristics of the participants is presented in Table 2. More than half (63%, 339/538) of the participants were in the late adolescent age (17–19 years) followed by the middle adolescent aged group. The minimum and maximum age of the participants was 14 and 19 years. The median age of the participants was 17 years with an interquartile range of 2 years. More than half (52.0%, 280/538) of the participants were female. More than half (64.1%) of the participants were from the nuclear family and lived with parents (83.8%, 345/538). Almost all participants' fathers (95.5%, 515/538) and mothers (92.6%, 498/538) had formal education. The respondents' fathers (78.4%, 421/538) and mothers (92.7%, 499/538) both had informal employment status at the data collection time.

**Table 2:** Sociodemographic characteristics of participants (*n* = 538).

**Characteristics**	**Frequency**	**Percentage**
Age*(***n*** **=** **538)**		
Middle adolescent (14–16 years)	199	37%
Late adolescent (17–19 years)	339	63%
**Sex (*****n*** **=** **538)**		
Male	258	48%
Female	280	52%
**Family type (*****n*** **=** **538)**		
Nuclear family	345	64.1%
Joint family	193	35.9%
**Living status (*****n*** **=** **538)**		
Parents	451	83.8%
Relatives	48	8.9%
Hostel	13	2.4%
Friends	9	1.7%
Alone	17	3.2%
**Father's education (*****n*** **=** **537)**		
Informal education	22	4.1%
Formal education	515	95.5%
**Mother's education (*****n*** **=** **538)**		
Informal education	40	7.4%
Formal education	498	92.6%
**Father's Occupation (*****n*** **=** **537)**		
Formal employment	116	21.6%
Informal employment	421	78.4%
**Mother's Occupation (*****n*** **=** **537)**		
Formal employment	39	7.2%
Informal employment	499	92.7%

* *Median (IQR) [Min, Max] 17 (2) (14, 19)*.

Table 3 shows the status of junk food consumption by school type. More than half of the participants of the public school consumed junk food (65.1%, 153/235), and the same was found among private school participants (56.3%, 161/286). Among the total participants, 60.30% (314/521) were junk food consumers as they consumed junk foods for four or more days last week. The remaining (39.7%, 207/521) were non-consumers that means they consumed junk foods for four or more days last week. Thus, higher junk food consumption status in public schools than in private schools was observed (65.1%, 153/235, 56.3%, 161/286, respectively).

**Table 3:** Status of junk food consumption (*n* = 521).

**Characteristics**	**School type**		**Total**
	**Public**	**Private**	
	**(*n* = 235)**	**(*n* = 286)**	
**Junk food consumption**			
Yes	153 (65.1%)	161 (56.3%)	314 (60.3%)
No	82 (34.9%)	125 (43.7%)	207 (39.7%)

Table 4 reveals the frequency and varieties of junk food consumption over the last month of data collection. Salty snacks were consumed by 59.8% (143/239) of participants from public school and 57.7% (165/286) from the private school. Similarly, sweet-related junk food consumption status, it was almost equal in both public and private schools (56.1%, 134/239 and 58.7%, 168/286, respectively).

**Table 4:** Average consumption of type of junk food in last month (*n* = 525).

**Characteristics**	**School type**		**Total**
	**Public**	**Private**	
	**(*n* = 239)**	**(*n* = 286)**	
**Type of junk food**			
Salty snacks	143 (59.8%)	165 (57.7%)	308 (58.7%)
Sweets	134 (56.1 %)	168 (58.7%)	302 (57.5%)
Sweetened beverages	101 (42.3%)	144 (50.3%)	245 (46.7%)
Fast food	76 (31.8%)	126 (44.1%)	202 (38.5%)
**Time of consumption**			
While with friend	197 (86.0%)	235 (82.2%)	432 (83.9%)
While traveling	166 (70.3%)	224 (78.3%)	390 (74.7%)
Special occasion	108 (48.0%)	175 (61.2%)	283 (55.4%)
While alone	108 (47.6%)	173 (60.5%)	281 (54.8%)
While with parents	103 (45.0%)	116 (40.6%)	219 (42.2%)
Not fixed time	65 (31.9%)	115 (41.4%)	180 (37.3%)
While reading	37 (15.7%)	40 (14.0%)	77 (14.8%)
**Place of consumption**			
On trip	147 (63.4%)	216 (75.5%)	363 (70.1%)
At restaurants	150 (64.9%)	190 (66.4%)	340 (65.8%)
At home	132 (57.1%)	171 (59.8%)	303 (58.6%)
At school	144 (61.3%)	154 (53.8%)	298 (57.2%)
At friend's home	84 (36.5%)	144 (50.3%)	228 (44.2%)
At street food stall	45 (19.6%)	77 (26.9%)	122 (23.6%)

The sweetened beverage was more prevalent at private school (50.3%, 144/286) compared with the public (42.3%, 101/239); the same result was found in fast food consumption status as well (44.1%, 126/286 and 31.8%, 76/239). More than three quarters of the participants from the public school (86%, 197/239) had a practice of consuming junk food with friends; however, participants from private schools (78.3%, 224/286) consumed the foods during travel time.

Occasional food consumption was found more in private school participants. However, they had more practice of taking junk food while they were being alone or while they were with their parents (60.5%, 173/286 and 40.6%, 116/286, respectively). This study also found that participants from public schools had more junk food consumption (15.7%, 37/239) at the time of reading, which was slightly less in participants from public schools (14.0%, 40/286). Consumption during the trip (75.5%, 216/286), at home (59.8%, 171/286), and with friends was more predominant with private school than with public school participants, while more proportion of public school participants had higher junk food consumption rate at a restaurant (64.9 %, 150/239) and at a school (61.3 %, 144/239).

Table 5 Depicts junk food consumption by some characteristics related to practice. Out of 522 participants, 39.3% (205/522) spent 0.85–2.5 US$ on junk foods followed by <0.85 US$ (27.8 %, 145/522), about 19.5% (102/522) spent 2.5 to 4$ and more than 4$ were expended by 13.4% (70/522) of the respondents. Junk food consumption by a family member was found to be 31.3% (163/522). A family member of private school participants had slightly more consumption (33.2%) than public school participants (28.9%). Nearly half of the participants (49.2%) consume junk food as an alternative to breakfast. Out of 522 participants, 38.9% (203/522) wanted to the junk food consumption. Similarly, out of 538 participants, 9.7% (52/538) mostly went outside of the home for dinner and had use of any item of junk food category.

**Table 5:** Distribution of weekly expenditure on junk foods, consumption accompanying fruits and vegetables among the participants.

**Characteristics**	**School type**		**Total**
	**Public**	**Private**	
**Weekly expenditure on junk food (*****n*** **=** **522)**			
<100 Rupees (<0.85$)	79 (33.5%)	66 (23.1%)	145 (27.8%)
100–300 Rupees (0.85–2.5$)	101 (42.8%)	104 (36.4%)	205 (39.3%)
300–500 Rupees (2.5–4$)	35 (14.8%)	67 (23.4%)	102 (19.5%)
>500 Rupees (>4$)	21 (8.9%)	49 (17.1%)	70 (13.4%)
**Junk food consumption accompanying with**			
Junk food eaten by family member (*n* = 163)	68 (28.9%)	95 (33.2%)	163 (31.3%)
Junk food eaten as alternative to breakfast (*n* = 257)	114 (48.3%)	143 (50.0%)	257 (49.2%)
Want to avoid junk food (*n* = 203)	91 (38.6%)	112 (39.2%)	203 (38.9%)
Go outside of home for dinner (*n* = 538)	19 (7.7%)	33 (11.3%)	52 (9.7%)
**Fruits and vegetables (*****n*** **=** **538)**			
1–2 times	59 (23.9%)	41 (14.1%)	100 (18.6%)
3–4 times	92 (37.2%)	91 (31.3%)	183 (34.0%)
5–6 times	52 (21.1%)	66 (22.7%)	118 (21.9%)
7 or more	44 (17.8%)	93 (32.0%)	137 (25.5%)

Furthermore, out of 538 participants, more than one third (34.0%, 183/538) of the participants had a practice of consuming fruits and vegetables three to four times a week. More than one fourth (25.5%, 137/538) of them consumed fruits and vegetables seven or more times, and one among five (21.9%, 118/538) consumed five to six times in a week. Only 18.6% (100/538) of them consumed fruits and vegetables one to two times a week.

Table 6 illustrates the knowledge level of junk food consumption and its consequences. Out of 538 participants, only 33.50% (180/538) had adequate knowledge regarding the harmful health effects of junk foods, and among them, more numbers were from private schools (37.5%, 109/291). Similarly, 66.5% (358/538) had inadequate knowledge of junk foods and its harmful effects. Among them, nearly three quarters (71.3%, 176/247) were from public school compared with private school.

**Table 6:** Knowledge on harmful health effects.

**Knowledge level (*n* = 538)**	**School type**		**Total**
	**Public**	**Private**	
	**(*n* = 247)**	**(*n* = 291)**	
Adequate	71 (28.7%)	109 (37.5%)	180 (33.5%)
Inadequate	176 (71.3%)	182 (62.5%)	358 (66.5%)

We compared the OR for the different sociodemographic, behavioral, and individual-level variables with junk food consumption (Table 7). It was found that participants of public school were 1.44 times more likely to consume junk foods. Similarly, children from a single family were 1.46 times, and those living with parents were 1.64 times more likely to consume junk foods. Time of consumption was explored and found, while on travel, 1.99 times, while reading 2.016, and while being alone, adolescents were 2.144 times likely to eat junk foods.

**Table 7:** Relationship of different sociodemographic, individual, and behavioral factors with junk food consumption.

**Characteristics**	**Consumption of junk food OR (95% CI)**
**School types**	
Public (Private = 1)	1.449 (1.015–2.068)
**Family types**	
Single (Joint = 1)	1.461 (1.014–2.106)
**Living condition**	
Parents (living with others = 1)	1.648 (1.031–2.636)
**Time of consumption**	
While travel (No = 1)	1.995 (1.335–2.982)
While reading (No = 1)	2.016 (1.169–3.474)
While being alone (No = 1)	2.144 (1.494–3.077)
Together with parents (No = 1)	1.540 (1.049–2.262)
No fixed time (No = 1)	1.614 (1.099–2371)
**Place of consumption**	
At home (No = 1)	2.206 (1.537–3.167)
At school (No = 1)	2.862 (1.988–4.121)
At restaurant (No = 1)	1.810 (1.249–2.623)
At trip (No = 1)	1.549 (1.056–2.271)
**Source of information**	
TV advertisement (rarely = 1)	OR 1.36(0.74–1.36)
Newspaper (rarely = 1)	1.601 (1.034–2.479)
Radio (rarely = 1)	OR 1.04(0.97–1.46)
Friends (rarely = 1)	2.011 (1.372–2.948)
Parents (rarely = 1)	1.500 (1.020–2.206)
**Availability of junk food at home**	
Yes (No = 1)	1.922 (1.338–2.762)
Junk food consumption by family member (No = 1)	1.617 (1.094–2.390)

*Reference, 1; OR, odds ratio; CI, confidence interval*.

Home and schools were more commonly observed places for junk foods with OR of 2.20 and 2.86, respectively. Among the sources of information, peer pressure was found to be more influencing, and they were likely to consume 2.01 while being with friends. Similarly, we also explored family member's roles. Those who reported the availability of junk foods at home were 1.92 times more likely to consume junk food.

## Discussion

In this study from Pokhara, we found that more participants were late adolescents, female, living in a nuclear family, and mostly living with parents. A higher number of fathers had received formal education than mothers and had a similar trend in employment status. Adolescents studying in public schools were consuming more junk foods than those in private schools. Salty snacks, sweets, sweetened beverages, and fast foods were frequently consumed junk foods. The time of consumption, traveling, special occasion, places of consumption, and weekly expenditure were explored in this study. We found that adolescents were also interested to avoid junk foods in their meals. More frequent (7+ times/week) consumption of fruits and vegetables was reported from private school participants. Inadequate knowledge of junk food and its long-term public health impact was found more common to participants from public school (71.3%,176/247) and private school participants (62 %,182/291), which suggests that more than half of the respondents had inadequate knowledge on junk food; thus, appropriate interventions need to be done to reduce consumption of such foods.

Consumption of junk foods and its association with different sociodemographic variables has been evaluated in our study; furthermore, published evidence supports that the dietary pattern and socioeconomic characteristics are associated (21, 25, 26). Similarly, consumption is also governed by availability and distance to junk food outlets (27, 28). The distance to the grocery store and fast food outlet is also found to be associated with skipping breakfast and free lunch at school and irregular eating habits (29). Furthermore, good taste, advertisement, easy availability of fast foods, and marketing are also found to be associated (30, 31). Other factors for growing fast food availability are increased earning, urbanization, busier lifestyle, fast service, assurance of food safety, and brands in China (32). Our study is of different nature, and we did not explore these factors; however, these might have definite impacts on the behaviors of the food of adolescents. One of such studies stated that there is no relationship with the proximity of restaurant and the body mass index (BMI) (33). Consumption of junk foods has been reported as risk factors for obesity and overweight among adolescents (34). More factors at the individual, social levels have a promotive role in fast food consumption in Teheran among adolescents (35). Advertisements and bored with family foods have been associated with fast food consumption (36). Our study shows similarities with the current research findings though we did not explore the BMI status and its factors for them.

Furthermore, adolescents living with parents are consuming junk foods more than others. Similarly, another research from the United States of America (USA) shows that those living with parents and in rented apartments have less frequent meals, poor dietary intake, and little home food availability compared with those living on campus (37). Moreover, another study from the USA shows that the food intake increased with increasing age and color of participants (38); however, we did not assess any role of ethnicity in this study, though our study population had a later adolescent aged population. In our study, we found that adolescents are more likely to consume junk food at home, schools, restaurants, and on a trip. Another study reveals that those taking lunch in the school canteen, hotels, and bakers are more likely to consume junk foods; parental influence on eating habits, eating dinner out, and consumption of vegetables and fruits have been found associated with junk food consumption (39). These factors reported from different studies were similar to our findings.

Furthermore, another research also highlighted the parental role in reducing the consumption of snacks high in solid oils, fats, and added sugars (SOFAS) (22). Our study demonstrated quite an interesting finding that adolescents living with parents also consumed more junk foods. However, friends were an important influencer to consume junk foods than parents. There are recommendations that the computation of fast foods have multiple factors including societal and individual level (35). Our study reported having similar elements in urban context of Pokhara, Nepal.

Increased fast food consumption is significantly associated with age, sex, family income, and residence (40). We also found an association with family types, family behaviors, and availability of foods. Since we conducted a study in urban settings, therefore, we are unable to comment on the difference on the basis of study settings, either urban or rural strata for food consumption. Another study from Pokhara shows that 75% of adolescents had good knowledge (15). However, in our study findings, 66.5% only had inadequate knowledge about junk foods. The varying proportion can be the used for the cutoff to define knowledge level. Furthermore, we only computed odds ratio without limiting other influencers. So, identification of the strongest influencer can be another scope of work. A study conducted on the general population in Singapore shows that regular fast-food consumers are those who are younger, belong to higher-income groups, and with middle-level education (41). Among the adolescents, there are various concerns related to foods and body images, dieting, education about foods, control of parents, educational level of mothers, and eating with family (42). In the present study also, we found positive association with some of these tested variables. In-depth exploring of these factors can be another sphere for the study.

Outcome of our study provides detailed understanding not only on knowledge, prevalence, and practice, influence of social media, peers, and family for junk food consumption among participants but also the reasons and influencing factors for participants to consume junk foods regardless of their knowledge on harmful effects and complications of junk food consumption. Similarly, this research work also provides a comparative insight information on junk food consumption pattern in public and private schools, which will be a supportive evidence for further policy implication.

Despite of these, our study is only limited to explore factors for junk food consumption among adolescents. We only relied on information given by them on the self-administered questionnaire. Therefore, we are unable to comment on the impact of these factors on their nutritional status due to lack of ABC parameters. These figures might have information bias, recall bias, copying other responses, and negligence to respond. We only recruited classes 11 and 12; therefore, junk food consumption status and other predictive factors of the other early adolescents might be missing. The economic status of the participants was not measured, although we supposed that it as an important factor. Junk food consumption is one of the growing concerns of the policymakers to safeguard public health globally. Therefore, we would like to recommend further study exploring ABC parameters of nutrition, their relationship with junk foods, frequency, lesser bias, and using the comprehensive technique of data collection.

Various cross-sectional studies have been conducted to assess the determinants of junk food and knowledge and practice of junk food consumption in different settings of Nepal and other low- and middle-income countries (LMICs), but our attempt to figure out public school and private school participants' junk food consumption status would be a further pathway to conduct comparative studies on similar topic to assess the health impact of junk foods among those who consume it and who do not consume it. Furthermore, comprehensive longitudinal studies will be a future direction to assess the growth and development of children and adults having junk food consumption practice.

Strategic risk communication to minimize junk food consumption should be prioritized, and interventions should be incorporated into national nutritional strategies. Behavior-change communication strategies should be tailored to targeted school children and general populations in order to address Nepal's food transition and long-term impact. We recommend further longitudinal research to assess epidemiological impact of junk foods, growth, and development of children and adolescents who had the history of regular junk food consumption.

## Conclusions

Our study findings reveal an increasing junk food consumption among school going-adolescents, which may contribute to poor growth outcomes. Consumption during travel time, restaurants, home, and school were found to be more common. Family and peer roles were also found to be more influencing for junk food accompanying the participants increased consumption. Interestingly, media exposure played a promotive role in junk food promotion, and among these, friend's influence is most influential.

Consumption of junk food among adolescent students was remarkably high in both public school and private school adolescents. Regardless of adequate knowledge on harmful consequences of junk foods, school-going adolescents are consuming junk food due to its easy availability and ready-to-use packaging. The government of Nepal should strictly standardize and regulate advertising policies and extravagant health claims advertised by junk food manufacturers. An appropriate intervention incorporated with national nutrition policies targeted to adolescents for improved food behaviors is recommended.

## Data Availability Statement

The raw data supporting the conclusions of this article will be made available by the authors, without undue reservation.

## Ethics Statement

The studies involving human participants were reviewed and approved by Pokhara University Institutional Review Committee(Ref # 28/074/75). Written informed consent to participate in this study was provided by the participants' legal guardian/next of kin.

## Author Contributions

SSB, KT, and LDB are principal investigators of this study, responsible for conceptualization, design, methodology application, data curation, data analysis, software application, writing an original draft, reviewing and editing, and overall supervision of the research. SSD and SW are responsible for the concept and design of the study, interpretation of the results, and preparation of the manuscript. LDB is responsible for conceptualization, design, methodology application, data collection, data curation, and analysis. All authors read and approved the final manuscript.

## Conflict of Interest

The authors declare that the research was conducted in the absence of any commercial or financial relationships that could be construed as a potential conflict of interest.
